# EDUCATIONAL APPROACHES TO FACILITATE LEARNING IN GERONTOLOGY AND GERIATRICS COURSES

**DOI:** 10.1093/geroni/igad104.1079

**Published:** 2023-12-21

## Abstract

Presenters in this session share teaching approaches designed to foster engagement and reduce barriers to students engaging in gerontology and geriatrics educational opportunities.

